# Untangling the influence of biotic and abiotic factors on habitat selection by a tropical rodent

**DOI:** 10.1038/s41598-021-91748-5

**Published:** 2021-06-18

**Authors:** Georgia Ward-Fear, Gregory P. Brown, David Pearson, Richard Shine

**Affiliations:** 1 School of Biological Sciences, Macquarie University, Office G17, Building 205B Culloden Road, Sydney, NSW 2109 Australia; 2 School of Life and Environmental Sciences , University of Sydney , Sydney, NSW 2006 Australia; 3 Western Australian Department of Parks and Wildlife, Waneroo, WA 6065 Australia

**Keywords:** Behavioural methods, Biological techniques, Ecology, Behavioural ecology, Community ecology

## Abstract

Understanding how animal populations respond to environmental factors is critical because large-scale environmental processes (e.g., habitat fragmentation, climate change) are impacting ecosystems at unprecedented rates. On an overgrazed floodplain in north-western Australia, a native rodent (Pale Field Rat, *Rattus tunneyi*) constructs its burrows primarily beneath an invasive tree (Chinee Apple, *Ziziphus mauritiana*) rather than native trees. The dense thorny foliage of the Chinee Apple may allow high rat densities either because of abiotic effects (shade, in a very hot environment) or biotic processes (protection from trampling and soil compaction by feral horses, and/or predation). To distinguish between these hypotheses, we manipulated Chinee Apple foliage to modify biotic factors (access to horses and predators) but not shade levels. We surveyed the rat population with Elliott traps under treatment and control trees and in the open woodland, in two seasons (the breeding season—January, and the nesting season—May). In the breeding season, we ran giving-up density experiments (GUD) with food trays, to assess the perceived risk of predation by rats across our three treatments. Selective trimming of foliage did not affect thermal regimes underneath the trees but did allow ingress of horses and we observed two collapsed burrows as a consequence (although long term impacts of horses were not measured). The perceived predation risk also increased (GUD values at food trays increased) and was highest in the open woodland. Our manipulation resulted in a shift in rat sex ratios (indicating female preference for breeding under control but not foliage-trimmed trees) and influenced rat behaviour (giving-up densities increased; large dominant males inhabited the control but not treatment trees). Our data suggest that the primary benefit of the Chinee Apple tree to native rodents lies in physical protection from predators and (potentially) feral horses, rather than in providing cooler microhabitat.

## Introduction

Animals typically use habitats non-randomly, and multiple factors influence their selection. Individuals trade off opportunities for reproduction, foraging, and safety, and those opportunities shift spatially and temporally in their environment^1,2^. Phenotypic variation in size, sex or temperament can also be influential: smaller animals may prefer more complex habitats for crypsis^3^; females may seek out certain substrates for nesting^4^; and bold animals may spend more time in predator-dense habitats^2^. Because habitat selection is behaviourally mediated, it is the primary mechanism that animals use to mitigate environmental stressors^5^. For example, prey species may adaptively alter their patterns of movement in response to real or perceived threats of predation^6^. Additionally, environmental perturbations (such as land clearing or climate change) can decrease the availability of suitable habitat by degrading the physical attributes of an environment or shifting abiotic factors that affect the ecology of many species^7^.

By influencing the distribution of faunal populations, these kinds of stressors can shape life history characteristics that are time and space dependent, such as social interactions and reproduction^8^. Ultimately, whether populations persist depends upon the nature of the stressor (and whether it is acute *versus* long term) and the ability of animals to facultatively adjust their patterns of habitat use^6^. To mitigate threats to wildlife, we must first understand how populations interact with their environments. Quantifying patterns of habitat use is straightforward but identifying the mechanisms that underlie habitat selection poses a formidable logistic challenge^9^. The fundamental difficulty lies in complex intercorrelations between potentially important axes of variation. For example, an animal may spend the day in a burrow rather than above-ground because the burrow is either cooler, or moister; because it protects it from predators, or it is defensible against conspecifics (or a combination).

Identifying the factors primarily responsible for driving non-random habitat use is challenging with correlational data alone^10,11^. To identify the proximate cues that animals use to select specific habitats, we need experimental manipulations to disentangle the impacts of correlated characteristics^12^. In many cases, such manipulations are difficult to conduct, especially under field conditions. In some cases, however, it is relatively easy to modify one suite of potential benefits to a specific microhabitat, while leaving other potential benefits unaltered. We exploited an opportunity of this kind to examine the mechanistic basis of habitat selection in a tropical rodent species (the Pale Field Rat, *Rattus tunneyi*) whose geographic distribution has shrunk by 85% in the last 200 years^13^. There is a wealth of studies on small mammals, generally, and on the genus *Rattus* specifically, looking at habitat use, social behaviour, population dynamics, and predator–prey relations. This background provides a good base from which to interpret the effects of habitat manipulations on rodent populations.

The Forrest River (Oombulgurri) floodplain in the east Kimberley region of north-western Australia experiences a severely hot climate, with air temperatures exceeding 30 °C year round (> 35 °C in 8 months^14^). During the long annual dry season (April to October), overgrazing by feral horses (which are now invasive in many areas of Australia^15^) creates an open landscape with little shade or shelter for native animals^16^. Our surveys showed that a native rodent (the Pale Field Rat, *Rattus tunneyi*) is found primarily in burrow systems beneath the wide-spreading, low-hanging, thorny foliage of the introduced Chinee Apple tree (*Ziziphus mauritiana*^16^). In a previous study on this system, we suggested two possible reasons for that strong association between the rat and the tree: abiotic—the trees offer a distinctive microhabitat that is shaded, and hence likely to be cooler than any other available areas, and biotic—the dense thorny foliage impedes ingress by horses (which would otherwise collapse burrows by trampling) and might also protect the rats from predation^16^. The physical structure of the Chinee Apple Tree is likely to prohibit predation by raptors, dingoes and potentially feral cats, and hinder predation by larger varanid lizards (but not snakes). In the current paper, we describe the results of a field manipulation in which we altered the structure of some Chinee trees (paired with control Chinee trees) designed to clarify the relative importance of abiotic and biotic factors in driving habitat selection by rats.

We predicted that if animals were selecting habitat in response to temperature, experimental manipulations that did not alter thermal profiles underneath trees would not affect the presence of rats. In contrast, if the rodents were responding to the risk of burrow damage by horses, we expected divergence in the use of treatment *versus* control trees by all rats inhabiting burrows, particularly a long time after the manipulation. Lastly, if rodents base movement and habitat selection predominantly on predation risk, we expected different cohorts and sexes to exhibit diverse patterns of habitat selection in response to our manipulations, that could reflect fear-thresholds of different cohorts within the rodent population.

## Methods

### Field site and study system

The 16,000 ha Forrest River Floodplain, in the east Kimberley region of Western Australia (15° 08′ 34 S, 127° 520′ 36 E) experiences a ‘tropical steppe’ climate, with high temperatures all year and highly seasonal rainfall (November to April, mean annual rainfall > 800 mm; *versus* May–October, mean annual rainfall < 50 mm^14^). Savannah woodland and short–tall grasslands dominate the floodplain and grade into spinifex grassland on top of the surrounding sandstone ranges.

The Pale Field Rat (*Rattus tunneyi*) is a nocturnal burrowing rodent endemic to Australia. Females reach maturity at approximately 60 g, and males at 90 g^13^. Primarily herbivorous, these rats excavate shallow burrow systems in loose substrate and are thought to live in extended social groups^17^. Similar to other rodents, individuals can be aggressive and territoriality can arise in either sex as a function of resource availability and population density^17,18^. Once widespread across mainland Australia, this species has been extirpated over more than 85% of its historic range and is declining in the remainder^13,17^. It is considered ‘Near Threatened’ nationally and listed as ‘Threatened’ in the Northern Territory^19,20^. Declines have been attributed to a combination of factors including habitat clearing^21^; overgrazing, soil compaction and burrow trampling caused by introduced species (rabbits, goats, cattle^22^); and predation by feral cats^23^. Native predators of Australian rodents include dingos, snakes, varanid lizards and raptors^13^.

The Chinee Apple tree or Indian jujube (*Ziziphus mauritiana*), native to tropical regions in Africa and Asia, has dense and highly spinose foliage. Mature trees typically exhibit an umbrella-shaped profile, up to 15 m tall and 20 m wide, with a narrow (often, < 30 cm) gap between the lowest foliage and the ground^24^. Hence, the area beneath this ‘umbrella’ is inaccessible to large animals. The species is thought to have been introduced to Australia by Chinese miners who cultivated it for its fruit (that is likely eaten by Pale Field Rats also^16^). The tree has spread throughout tropical regions including north-western Australia and is a declared pest in Western Australia^25^. Our surveys on the Forrest River floodplain showed that although Chinee Apple trees comprised only 9% of woodland trees, they accounted for 58% of all trees with rodent burrows beneath them^16^.

### Foliage-manipulation experiment

#### Manipulation

To clarify causal mechanisms underlying the preferred association of rats with Chinee Apple trees, we first selected 10 pairs of Chinee Apple trees growing < 30 m apart and matched for size. All trees had rodent-burrow systems under them, and adjacent sites were separated by 100–200 m. We removed the lower foliage branches to a height of 3 m up the trunk from one tree of each pair (the ‘trimmed’ tree). The tree thereafter retained its umbrella shape, but with a higher gap between the lowest foliage and the ground. The foliage of the other tree of each pair was left intact, such that thorny branches were present almost to the ground level, creating the typical dense umbrella form. The manipulation was designed to allow easier access to dingos, raptors, large varanid lizards and feral cats.

#### Monitoring the effects of manipulation

We buried thermal-logging ibuttons (Maxim, Thermodata Pty. Ltd.) 20 cm underground at the base of five pairs of trees (n = 10 trees total) and recorded temperatures at hourly intervals for eight days. Data on soil temperatures were pooled into three-hourly means and temporal changes between trimmed and intact trees were compared using a repeated-measures ANOVA. Because the temperature data violated the assumption of sphericity, we used the Greenhouse–Geisser (G–G) correction to adjust the degrees of freedom and F test^26^.

We trapped small mammals under the 10 pairs of trimmed and intact Chinee Apple trees, once in the wet season (January, three months post-manipulation) and once in the dry season (May, seven months post-manipulation). At these times, any regrowth was removed from the trimmed trees. An Elliott trap (Elliott Scientific Equipment, Upwey, Vic) containing 20 g of peanut butter and raw rolled oats was placed at the base of each ‘trimmed’ and ‘intact’ Chinee Apple tree. Another trap was placed in the ‘open woodland’ (no direct canopy cover, and grass < 20 cm high), midway between the trimmed and intact trees. Trapping sessions ran for five nights; traps were left open each night and were checked at dawn, then remained closed from dawn until dusk. For each small mammal we recorded trap location (trimmed tree, intact tree, open woodland), mass, sex, age class (adult or juvenile based on size and visual evidence of sexual maturity) and, in adults, current reproductive condition (reproductive: males have enlarged distended testes, females have bare, elongated teats > 2 mm^17^). We marked the tail of each rat with a unique ID mark using a non-toxic permanent marker so that we could quantify rates of recapture and movement within the trapping session.

We ran a full factorial, multiple logistic regression to determine whether treatment or season (or treatment × season) had the strongest influence on whether or not a trap captured a rodent (dependent variable: capture y/n) and also the sex of the rodent captured (dependent variable: sex) Then, to explore how the rodents had reacted to our habitat manipulations in each season, we ran analyses with ‘Treatment’ (trimmed tree, intact tree, open woodland) as the independent variable, and life history (age class, reproductive status), morphology (mass) and trapping data (number of captures and sex) as the dependent variables. For analyses that had a categorical binomial dependent variable (e.g., sex, age class) we ran Generalised Linear Mixed Models with a binary distribution and a logit link function in SPSS version 25 (IBM Institute). To analyse rodent ‘mass’ across treatments, we looked at the sexes separately using ANOVAs in JMP 14.1 (SAS institute, Cary NC). In all analyses, we included ‘trap site’ as a random factor to account for repeated measures.

To assess whether our foliage manipulation affected access by horses, we recorded all sightings of horses, new dung piles or hoof prints directly underneath the canopy ‘footprint’ of both trimmed and intact trees. We ran these surveys in January, as we visited our traps each day, both in the morning to check and close traps, and again in the afternoon to open and rebait traps. To ensure we did not scare animals away before recording them, we looked for horses under the trees at the next site (i.e., from 100 m away) through binoculars, before approaching for trap maintenance. We compared the incidence of this ‘horse presence’ using Chi-square analysis.

#### Giving-up densities

Using depletable food patches, we conducted giving-up density (GUD) experiments^27^ in January (the wet season, when rats are reproductive), to quantify rodent perceptions of nocturnal risk under intact trees versus trimmed trees versus open woodland. We ran this trial a week after our rodent surveys had ceased so as not to confound trapping rates. We mixed 12 quarter pieces of unsalted peanuts in 125 ml of clean river sand, in a plastic container (20 × 12 × 2 cm) and buried the container such that the top was level with the ground surface. We placed one of these containers under each of our ‘intact’ and ‘trimmed’ trees (50 cm from the trunk) and at the ‘open’ trapping location, at each of our 10 sites. Containers were set out at dusk; the following morning at dawn, we sieved the sand and counted how many pieces of peanut were left in each container. The premise of GUD experiments is that the amount of time an animal spends foraging is inversely related to the perceived risk of predation in that microhabitat, such that more food will remain in containers in areas of higher perceived risk^27,28^ (but see Bedoya-Perez et al.^29^ for discussion and limitations). The trial ran for three nights, and we deployed new sand and peanuts each day. We set remote cameras on a random sample (n = 10) of our GUDs across treatment and location to confirm the identity of foragers. To look at variation in GUDs across different environments, we ran a full factorial Generalised Linear Mixed Model with Poisson distribution and logit link function. The independent variables were treatment, site and the interaction term, treatment X site. The ‘number of peanuts left at trays’ (i.e., the giving-up density) was the dependent variable. We included the unique location of each GUD tray as a random factor to correct for repeated measures. Tukey post-hoc tests were then used to identify differences between treatments. Analyses were conducted in SPSS version 25 (IBM Institute). The outputs of all statistical tests are collated in Appendix Table 1.

#### Ethics statement

Ethical protocols were approved by the University of Sydney’s Animal Ethics Committee (AEC protocol: 2103/6034; DPaW permit: SF010079) in accordance with the international ‘Principles of Laboratory Animal Care’.

## Results

### Temperature

**Figure 1:**
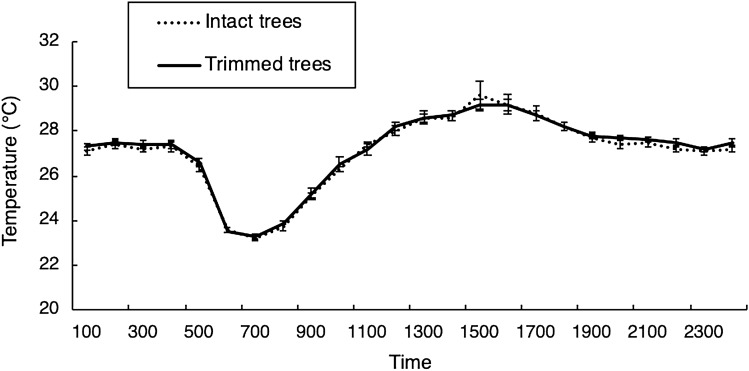
Hourly soil temperature measured under Chinee Apple trees with lower foliage removed (‘Trimmed’ n = 5) *versus* those that were left ‘Intact’ (n = 5). Temperatures were recorded over eight days during January. Data were pooled into three-hourly categories for analysis. Mean temperature changed significantly with time but did not differ between trimmed vs intact trees. Hourly temperatures are represented as the mean across all sites under either trimmed or intact trees, with error bars of one standard error from the mean.

Removing the lower foliage branches of a Chinee Apple tree did not affect the thermal environment beneath its foliage. Although temperature changed substantially with time of day (G–G F_2,16_ = 219.4, *p* < 0.001), there was no significant thermal difference between trimmed or intact trees (‘treatment’) at any time of day (treatment effect: G–G F_2,16_ = 0.29, *p* = 0.76; treatment*time of day: G–G F_2,16_ = 0.29, *p* = 0.76, Fig. 1).

### Rodent trapping

We captured 41 individual animals in the January wet season trapping session (open woodland 15, trimmed trees 12, intact trees 14) and 42 individual animals in the May dry season trapping session (open woodland 7, trimmed trees 16, intact trees 19; See Appendix Table 2 for trapping data). The interaction between trapping session and treatment was significant (χ^2^ = 9.41, df = 2 N = 281, *p* = 0.009; recaptures excluded from this analysis). In the first trapping session, equal numbers of individuals were caught across all treatments, whereas in the second trapping session significantly fewer animals were found in the open woodland.

Overall, treatment was the strongest determining factor for sex ratios across both trapping sessions (χ^2^ = 6.7, df = 2 N = 101, *p* = 0.03). For many relationships there were strong seasonal differences, reflecting the progression of annual activity and life history events within the rat population; we describe them separately below.

**Figure 2:**
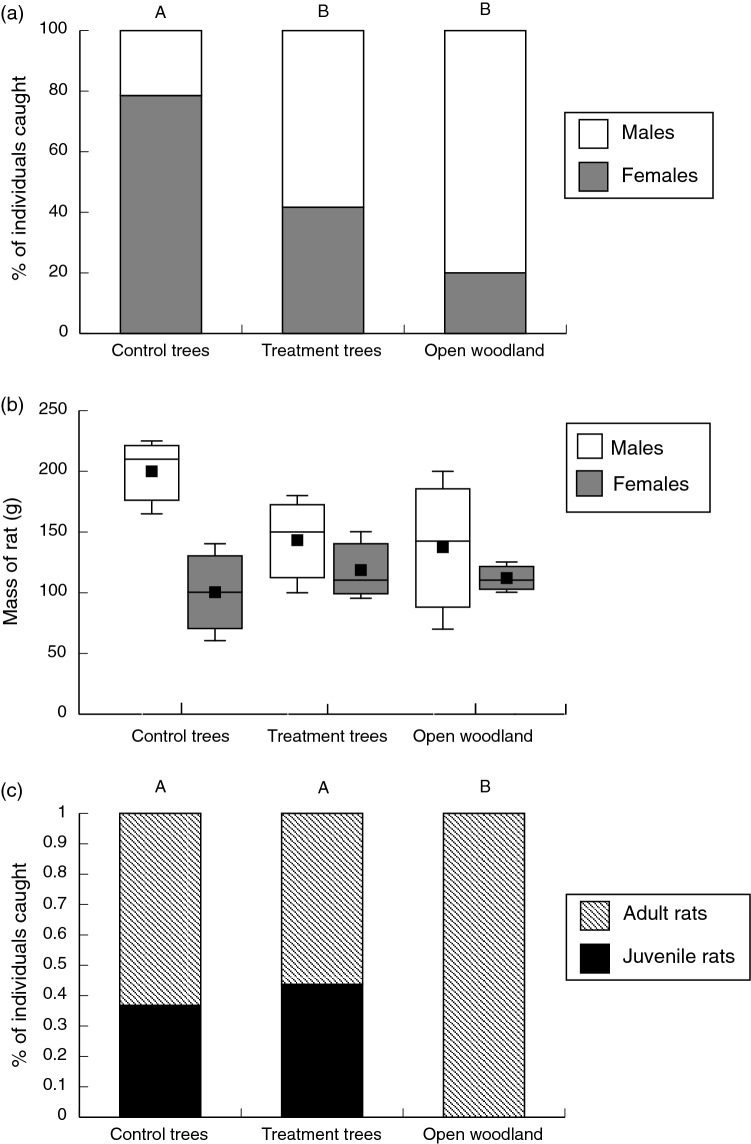
Effect of habitat on ecological traits of Pale Field Rats. Animals were trapped in ‘Open Woodland’ or under Chinee Apple Trees with intact foliage (‘Intact trees’) or with lower foliage removed (‘Trimmed trees’). Bars denoted by the same letter within each panel are not significantly different (*p* < 0.05). (**a**) Sex ratios during the January breeding season. (**b**) Body mass of each sex during the January breeding season; box and whisker plots denote mean, median, minimum, maximum, and interquartile ranges. (**c**) Age structure (adults vs. juveniles) during the May nesting season.

In the wet season, we captured only sexually mature adult rats (19 F, 22 M), all of which were reproductive. Thus, we considered this the breeding season for Pale Field Rats^13^. The relative numbers of male and female rats differed among intact trees, trimmed trees and open woodland (F_2,32_ = 3.83, *p* = 0.032; Fig. 2a). Female rats were more common under the intact trees, followed by the trimmed trees; few females were caught in the open woodland. Conversely, male rats were most common in the open woodland, followed by the trimmed trees and then the intact trees. The males found under intact trees (where the most females were found) were heavier than those found under the trimmed trees or in the open woodland (F_2,17.66_ = 4.04, *p* = 0.036, Fig. 2b). The mean mass of female rats did not vary significantly among the three locations (F_2,13_ = 0.89, *p* = 0.43). There were no recaptures during this trapping session.

In the dry season we caught both adult and juvenile rats (26 Female, 16 Male; 28 Adult, 14 Juvenile), and considered this the nesting season for Pale Field Rats^13^. The recapture rate was 30% under both intact and trimmed trees (tree comparison *p* > 0.8), and 0% in the open woodland. All recaptures were found at the same trap location (i.e., we did not document movement of any individual between individual trees, or sites). At this time of year, experimental treatment had no significant effect on the sex ratio or mean mass of adult rats (all *p* > 0.5), although the same trends as observed during the wet season (January; see above) were apparent. However, location did influence the ratio of adult to juvenile rats caught (adult rats were caught in relatively equal numbers at all locations, whereas juvenile rats were never caught in the open woodland: Fig. 2c).

### Giving-up densities

**Figure 3:**
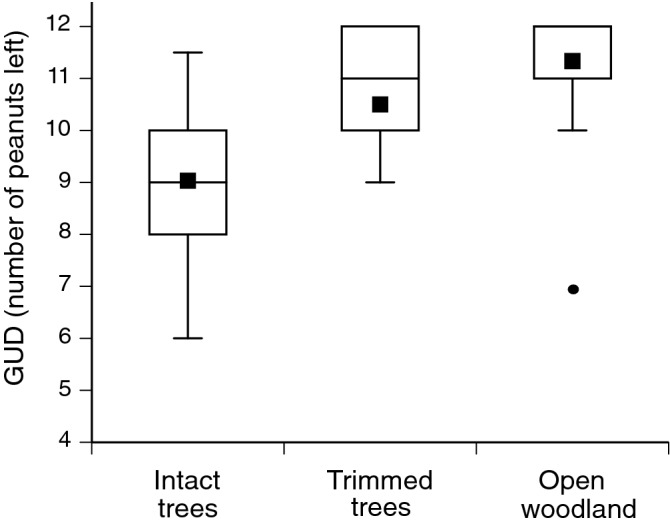
Results from giving-up density (GUD) experiments conducted in January to measure ‘perceived risk’ by rats foraging under ‘Intact’ Chinee Apple trees (with intact foliage), ‘Trimmed’ trees (lower foliage removed) or ‘Open woodland’. The GUD is expressed as mean number of peanut pieces (out of 12) remaining after nocturnal foraging activity. Higher GUD values indicate higher perceived risk and increase with less cover close to the ground (i.e., structural openness of habitat: open woodland > trimmed > intact). Box and whisker plots denote mean, median, minimum, maximum, and interquartile ranges. Plots may have no upper whisker when the upper interquartile range is the same value as the sample’s maximum. All treatment categories were significantly different from each other (*p* < 0.05).

Tracks, droppings and remote camera photos demonstrated that our food-trays were visited exclusively by Pale Field Rats. The interaction between site and treatment was not significant, GUDs differed across Treatments (F_2,60_ = 3.81, *p* = 0.028; Fig. 3) and this did not change between sites. Tukey post hoc tests revealed that each location differed significantly from the others. GUDs were lowest at the intact Chinee Apple trees (i.e., the most food was eaten) followed by the trimmed trees and then the open woodland (i.e., the least amount of food eaten). We did not witness more than one rat visiting a food tray at any one time.

### Access to horses

We recorded horses and/or their dung under 6 of our 10 trimmed Chinee Apple trees post-thinning, but not under any intact trees (n = 11 sightings of horses, n = 4 sightings of dung). Thus, our manipulation enhanced access by horses (χ^2^ = 10.97, n = 20, *p* < 0.001). The areas beneath these trees exhibited varying levels of soil compaction and in two cases, partial burrow collapse due to horse activity (i.e., horse-hoof shaped holes caving burrows in).

## Discussion

A relatively minor alteration to the environment elicited significant shifts in the behavioural patterns of resident Pale Field Rats, and thus their demographic spread across the landscape. These shifts were not due to changing thermal regimes (temperatures across locations remained the same), but instead were responses to increased structural ‘openness’ of the habitat; changes that may have resulted in increased exposure to predators and potential damage by feral horses.

Habitat degradation is a common consequence of invasive ungulates worldwide^30^. Overgrazing and trampling are key impacts of horses in Australia, and can influence populations of small mammals^15^. Kutt and Gordon^31^ found differences in small mammal diversity and abundance between grazed and ungrazed areas, and Legge et al.^32^ noted trampling and soil compaction as a potential effect on populations of small mammals in tropical Australia. In montane regions, overgrazing by feral horses changes habitat composition and structural complexity, exposing broad toothed rats (*Mastocomys fuscus*) to higher rates of predation and leading to their extirpation from areas with high abundance of horses^33,34^. In keeping with this broader background, previous studies have speculated that hoofed ungulates can impact populations of *Rattus tunneyi* via refuge destruction^16,35,36^.

In our experiment, removing the lower foliage of trees allowed horses to access the area around the trunk and consequently, two rat burrow systems were damaged. Furthermore, when horses repeatedly seek shade under trees, compaction by these heavy mammals changes soil characteristics^16^ and over time, it would likely become increasingly difficult for rodents to maintain their loose, shallow burrows^13^. Although we witnessed horses damaging burrows, we did not measure the impact of horses rigorously enough to draw strong conclusions about their influence on rodent habitat selection over the long term. If the risk of horse damage was the main driver of habitat selection by rodents in our study, we would have expected all rodents (irrespective of sex or age) to avoid trimmed trees—especially in the dry season sample, when horse damage would have accumulated over a long period. Nevertheless, our short-term study was able to document feral horses damaging the burrow systems of a threatened Australian rodent, and this suggests that the phenomenon is much more pervasive than reported in literature. Our findings support the push by conservation managers to remove feral horses from Australian ecosystems.

Predation risk may have been a greater influence on microhabitat selection by rodents than was trampling by horses, for reasons we now discuss. The ‘landscape of fear’ theory conceptualises the area used by a prey species into patches of microhabitat embedded in a wider landscape; patches are high- or low-risk depending on the abundance and lethality of predators within them^37^. For example, rodents in predator-free enclosures use spaces away from shrubs more often and create more (and less straight) runways throughout their home ranges^38^. GUD experiments can provide a measure of the ‘perceived cost of foraging’ (and thus, fear) for prey species in any given patch^27,28^ (but see Bedoya-Perez et al.^29^). An animal’s vigilance will scale with habitat complexity (or refuge components therein^39^) depending on the hunting style of the predator it is evading^40,41^.

Many predatory species pose a threat to Pale Field Rats^13^; during this study alone we recorded predation on rats by elapid and pythonid snakes, varanid lizards, dingoes, feral cats and several raptor species (GWF, unpubl. data). The physical structure of the Chinee tree hinders access to many (but not all) of these species. Predictably, rats spent the most time foraging under the densest vegetation of intact trees, as indicated by the lowest GUDs. Removing the lower foliage of the trimmed trees did not cause widescale departure of rats but did appear to increase the perceived risk of predation and created a level of GUD intermediate between the open woodland (highest risk; shortest time spent foraging; highest GUD) and the intact trees (lowest risk; longest time spent foraging; lowest GUD). This GUD response suggests that our manipulation altered habitat quality for rats with respect to predation. That is, at trimmed trees, rats displayed increased nocturnal vigilance and antipredator behaviours without a significant decrease in their numbers. It is important to note, however, that many factors can influence GUD results and confound simple interpretations, such as differences in absolute rat abundance, sex or age; animal foraging styles (group *versus* solo foraging); or the energetic states of consumers^29,42^. Such factors are difficult to control in the wild. For example, although we never witnessed rats group-foraging, this behaviour may occur. Likewise, we did not find significant differences in (captured) rat numbers between sites, but such differences may be present. The GUD values that we documented conformed to the patterns based on prey vigilance under different levels of structural habitat openness, but we cannot definitely prove that causation.

In tropical savannahs, even low densities of feral cats can substantially impact populations of small mammals over short periods^43^. In a recent study, two cats killed 17 of 18 individuals in a population of Pale Field Rats^23^. In another Australian study, the removal of undergrowth (by fire) led to a five-fold increase in predator abundance and a subsequent decline in the number of *Rattus fuscipes*^44^, and predation by native and feral predators can curb irruptive population events in rodents^45^. Although we did not measure predator abundance directly, the large body of evidence that relates habitat complexity with predation risk for small mammals suggests that our manipulation may have increased the risk of predation for *R. tunneyi* at those sites.

An animal’s response to habitat treatment was strongly influenced by its sex. We speculate that the habitat preferences of adult females likely drove the wider shifts in spatial ecology, social dynamics and age structure that we saw. In small mammals, the abundance, renewability and distribution of resources influence female reproductive success, and territoriality^46,47^. Herbivorous species that utilise fast-growing fruits and grasses exhibit low territoriality, such that the distribution of females mirrors the distribution of resources (both food and habitat) across the landscape, rather than being driven by intraspecific aggression. When high-quality resources are patchy or limited, females can ‘clump’ throughout the landscape^18^; this distribution increases the payoffs for territoriality in males^48^. Our results accord with this scenario. By increasing the risk of predation under some trees but not others (i.e., limiting the availability of a high-quality resource [a safe refuge]), we condensed female distribution into pockets around the best-quality habitat (intact trees), and males responded accordingly. This effect was strongest during the breeding season; only a few adult males inhabited the area underneath the intact trees (that sheltered most of the females). These males were larger than males in other areas, suggesting dominance and territoriality^49,50^. Although little is known about the behaviour or social structure of *R. tunneyi*^36^, the honing of social hierarchy in response to resource quality (which may or may not relate to predation risk) is common in the *Rattus* family, many species of which are polygynous, with increased male size equating to dominance^51^. In May, after competition for mates had subsided, the effects of our experimental manipulation on sex ratios and mass of adult rats diminished (although the same trends remained). Although we did not monitor horse impacts closely enough to fully clarify their influence on the rats, we speculate that if rodents been reacting to the risk of habitat degradation wrought by horses (or even the act of manipulating the habitat of and in itself), it is likely that both sexes would have responded similarly to the treatments. Additionally, we may have seen decreases in rat numbers under the trimmed trees over time (i.e., in the dry season session, as rodents moved away from horse prone areas after prolonged exposure). Instead, it’s likely that behavioural attributes (such as boldness) vary between the sexes in ways that influence the strength of their antipredator responses and subsequent habitat selection^50^.

Despite female rats preferring intact trees during the breeding season, some nesting did occur in burrows beneath the trimmed trees as well. This could reflect space-limitation under intact trees, less competitive females being relegated to lower quality habitat, or a seasonal shift in the quality of resources (such as fruit load on trees) that we did not measure. The open woodland, however, is an exposed, risky place for a small rodent year-round (as evidenced by high values of GUD). Here, we caught mostly males (no juveniles, few females and no recaptures), mostly in the breeding season and likely just dispersing through the woodland (as no burrow systems were found in these areas^16^). As a consequence of life history strategies and behavioural differences between the sexes^50^, male rats are likely trading off predation risk with access to feeding or mating opportunities. In this system, physical features of the environment shape social interactions within a population. By altering components of habitat, we manipulated how animals were spread across the landscape, and thus, their intraspecific interactions. Our findings support the idea that movement of individuals can mediate patterns of social interactions, and thus wider social organisation in group-living species^8^.

Environments in which prey animals are physically exposed become high-risk areas in their landscape of fear^52^. Prey species respond rapidly to alterations in habitat or predator guilds, and sometimes this can entrain wider ecological processes. For example, the reintroduction of wolves into Yellowstone National Park changed patterns of aggregation, vigilance and habitat selection of elk^53^. But those antipredator behaviours imposed physiological costs on the elk which impacted calf fitness. Thus, an apex predator indirectly regulated the reproductive physiology of its prey^53^. Similarly, in our study, we speculate that the risk of predation altered the spatial ecology and thus social dynamics of the rodent population. This in turn could influence which individuals in the population bred successfully. Broadly, any shift in density, sex ratios or mating systems can influence effective population sizes, and hence the long term genetic diversity and viability of small populations^54,55^. Our manipulation demonstrates the speed with which resident population structures can change following habitat modification.

Another plausible factor influencing habitat use by the rats in the woodland could be the large palatable fruits produced by Chinee Apple trees^16^. In tropical savanna *R. tunneyi* are mainly ‘grass specialists’, eating a substantial amount of fresh grass stems, seeds and roots^56^. Nonetheless, seasonal fruit may be an important part of the diet for *Rattus* sp. (and for pregnant and lactating females, in particular^51^). These trees hence may provide both cover and an important food source at certain times of year^16^. However, removing the lower branches of Chinee Apple trees is unlikely to substantially decrease the amount of fruit available to the rats (when it ripens in the dry season). Thus, we doubt that reduction in fruit supply drove the shifts in habitat use by rodents during the wet season (when no fruit was present). Instead, the availability of fruit may contribute to the rodents’ overall preference for Chinnee trees *versus* other native woodland species^16^.

An interesting qualifier to this study is that the Chinee Apple tree is invasive. In degraded systems, invasive species can play positive ecological roles via ‘ecosystem engineering’^57–59^. However, few studies have quantified how the demographic and behavioural traits of native populations respond to the habitat modifications wrought by invaders (but see^59–61^). Our study demonstrates the facilitative role that the Chinee Apple tree plays for the Pale Field Rats, in a system facing multiple threats; indeed, the rats may not persist there without them^16^.

Although targeted habitat restoration with native species can help to restore biodiversity and natural ecosystem function in degraded environments^62,63^, this is not feasible everywhere. Our field site is remote and there is no native equivalent to the Chinee tree that can match the resource production to support rodents there, given the compounding threats (for comparison of tree species see^16^). Our study reinforces a growing conclusion: land managers should view introduced species in a framework of ecological functionality^57^. An empirical assessment of trophic networks within the environment can elucidate whether the invader’s removal will imperil the persistence of other native species^57,58^.

In conclusion, our manipulative study demonstrated the influence of biotic and abiotic parameters on habitat selection in a small mammal. A superficially minor manipulation of habitat evoked changes in the spatial ecology, social systems and population demography of rats. We speculate that this was as a response to altered breeding opportunities and an increased perceived risk of predation. Facultatively modifying habitat use as a first-line response to perturbations may be effective for individuals in the short term, but large or prolonged shifts in spatial ecology (and thus, social organisation) can have deleterious consequences for the viability of a population in the long term^64^. Future research could usefully monitor these populations over the long term, to clarify the possible influences of interannual changes in environmental conditions and/or fluctuations in rodent numbers. Nevertheless, our case study has broader implications. Large-scale environmental degradation is impacting ecosystems at unprecedented rates^65^, sometimes forcing native species to rely on invasive ecosystem engineers to maintain ‘ecological handholds’^16^. Untangling the proximate mechanisms of habitat selection is the first step to developing threat mitigation strategies for such at-risk taxa.

## Supplementary information

Supplementary Informations.
