# Supplementary material for: Untangling the influence of biotic and abiotic factors on habitat selection by a tropical rodent
Source: Sci Rep. 2021 Jun 18;11:12895. doi: 10.1038/s41598-021-91748-5 (PMC8213835; doi:10.1038/s41598-021-91748-5)
Supplement: Supplementary file 1 — Supplementary Informations. [file 41598_2021_91748_MOESM1_ESM.docx]

**Appendix**

**Appendix Table 1:** Summary of statistical outputs from various analyses, and their interpretations. Abbreviations are as follows: Treatment (Tr): Intact Tree (IT), Trimmed Tree (TT), Open woodland (O). Season (S; Breeding and Nesting), Female (F), Male (M). Where relevant, the test statistic, degrees of freedom and sample size have been provided. P values are denoted in italics and all significant relationships have been highlighted with bold text (in both the statistical and conclusion column).

| **Test variables** | **Statistical details** | **Conclusion** |
| --- | --- | --- |
| Horse sign presence/absence  Trimmed vs. Intact trees | χ^2^ = 10.97, N= 20  ***0.0009*** | **TT > IT** |
| Hourly temperature:  Trimmed Trees vs Intact Trees | G-G F_2,16_ = 0.29  *0.760* | TT = IT |
| Overall influence of Treatment vs Season  on rate of captures | χ^2^ = 9.41, N= 281, P ***0.009*** | **Tr * S**  **Jan: IT = TT = O**  **May: IT = TT > O** |
| Overall influence of Treatment vs Season  on sex of captures | χ^2^ = 6.7, N= 101  ***0.03*** | **Tr > S** |
| Breeding season:  sex ratios across treatment | F _2,32_= 3.83,  ***0.030*** | **F: IT > TT > O**  **M: O > TT > IT** |
| Breeding season:  mean mass of rats across treatment | F: F _2,13_= 0.89  *0.43*  M: F _2,17.66_= 4.04  ***0.036*** | F: IT = TT = O  **M: IT > TT > O** |
| Nesting season:  sex ratios across treatment | F _2,30_= 0.68  *0.51* | F: IT = TT = O  M: O = TT = IT |
| Nesting season:  mean mass of rats across treatment | F _2,16.92_= 0.35  *0.71*  M: F _2,6.67_= 4.51  *0.06* | F: IT = TT = O  M: IT = TT = O |
| Giving Up Density values across treatments | F_2,60_ = 3.81  ***0.028*** | **O > TT > IT**  High value = less foraging |

**Appendix Table 2:** Capture data for rodents by sex and trap day in different treatments across the wet season trapping session and the dry season trapping session.

| Trapping session | Trap day | Intact trees | |  | Trimmed trees | |  | Open Woodland | |  |
| --- | --- | --- | --- | --- | --- | --- | --- | --- | --- | --- |
|  |  | Male | Female | Total | Male | Female | Total | Male | Female | Total |
| Wet season (Jan) | 1 | 0 | 1 | 1 | 1 | 0 | 1 | 1 | 1 | 2 |
| Wet season (Jan) | 2 | 0 | 2 | 2 | 3 | 2 | 5 | 1 | 0 | 1 |
| Wet season (Jan) | 3 | 2 | 3 | 5 | 1 | 1 | 2 | 4 | 0 | 4 |
| Wet season (Jan) | 4 | 1 | 4 | 5 | 2 | 2 | 4 | 4 | 1 | 5 |
| Wet season (Jan) | 5 | 0 | 1 | 1 | 0 | 0 | 0 | 2 | 1 | 3 |
| **Session total** |  | 3 | 11 | **14** | 7 | 5 | **12** | 12 | 3 | **15** |
|  |  |  |  |  |  |  |  |  |  |  |
| Dry season (May) | 1 | 3 | 3 | 6 | 2 | 3 | 5 | 1 | 0 | 1 |
| Dry season (May) | 2 | 2 | 3 | 5 | 1 | 4 | 5 | 0 | 0 | 0 |
| Dry season (May) | 3 | 1 | 3 | 4 | 4 | 3 | 7 | 1 | 0 | 1 |
| Dry season (May) | 4 | 2 | 3 | 5 | 1 | 3 | 4 | 0 | 1 | 1 |
| Dry season (May) | 5 | 3 | 6 | 9 | 1 | 2 | 3 | 2 | 2 | 4 |
| **Session total** |  | 11 | 18 | **29** | 9 | 15 | **24** | 4 | 3 | **7** |
